# Rare disease genomics and justice: overview of a workshop at the Fondation Brocher, 22–24 January 2025

**DOI:** 10.1007/s12687-026-00911-w

**Published:** 2026-07-01

**Authors:** Angus Clarke, Ruth Horn, Elena Avram, Siddarth Banka, Felicity Boardman, Victoria Charlton, Mike Drummond, Roseline Favresse, Angeline Ferdinand, David Freis, Ilaria Galasso, Nadine Z. Großmann, Meghan Halley, Sasha Henriques, Hadewych Honné, Sofia Douzgou Houge, Salman Kirmani, Tamar Nov Klaiman, Teodora Lalova-Spinks, Charlotta Ingvoldstad Malmgren, Nick Meade, Elena Nicod, Kelly E. Ormond, Barbara Prainsack, Hilde Van Esch, Ambroise Wonkam, Sarah L Wynn, Francesca Forzano

**Affiliations:** 1 Cardiff University, Wales, UK; 2 University of Augsburg, Augsburg, Germany; 3 Medlife Clinics, Bucharest, Romania; 4 Division of Evolution and Genomic Sciences, School of Biological Sciences, Faculty of Biology, Medicine and Health, Manchester Centre for Genomic Medicine, Health Innovation Manchester, University of Manchester and, St Mary’s Hospital, Manchester University NHS Foundation Trust, Manchester, UK; 5 University of Warwick, Coventry, England UK; 6 London School of Economics, London, England UK; 7 University of York, York, England UK; 8 EURORDIS, Paris, France; 9 University of Melbourne, Victoria, Australia; 10 University College Dublin, Dublin , Ireland; 11 FOP e.V., International FOP Association (IFOPA), Freie Universität, Berlin, Germany; 12 Center for Biomedical Ethics, Stanford University, Stanford, CA USA; 13 Genomics England, London, UK; 14 University of Edinburgh, Edinburgh, Scotland UK; 15 Leuven, Belgium; 16 Haukeland University Hospital, Bergen, Norway; 17 The Norwegian Centre for Rare Diseases, Oslo, Norway; 18 Medical Genetics and Paediatric Endocrinology, Medical College, The Aga Khan University, Karachi, Pakistan; 19 Department of Public Health and Primary Care, Faculty of Medicine and Health Sciences, Ghent University, Ghent, Belgium; 20 Centre for IT and IP Law (CiTiP), Faculty of Law, Ghent, Belgium; 21 Clinical Pharmacology and Pharmacotherapy, Department of Pharmaceutical and Pharmacological Sciences, Faculty of Pharmaceutical Sciences, KU Leuven, Ghent, Belgium; 22 Center for Rare Diseases, Department for clinical genetics, Karolinska University Hospital, Stockholm, Sweden; 23 Center for Research Ethics and Bioethics, Uppsala University, Uppsala, Sweden; 24 Genetic Alliance UK, London, England UK; 25 Dolon Ltd., London, UK; 26 ETH-Zürich, Zürich, Switzerland; 27 University of Vienna, Vienna, Austria; 28 Johns Hopkins University School of Medicine, Baltimore, Maryland USA; 29 UNIQUE, London, England UK; 30 Guys and St Thomas’ NHS Foundation Trust and Faculty of Life Sciences, King’s College London, London, UK

## Abstract

Genomics is transforming health care but its implementation raises challenges. This paper reports a 2025 workshop on justice in the implementation of genomics for rare disorders. The workshop goals were to develop a consensus understanding of the problems faced by rare disease patients and families where justice is at stake, to achieve a shared perspective on support for rare disease patients, and to consider the implications for justice in several areas of rare disease genomics, in both research and healthcare. We heard about the diverse experiences and needs of patients. Inequity between different rare diseases is marked. The need for coordination of care for rare disease patients is under-recognized but good models of rare disease care exist. The value of conscientious professionalism to nurture a rare disease mindset needs to be emphasized in the training of each new generation of healthcare students//trainees. The circumstances of different population groups differ systematically. The needs of indigenous and other historically marginalised groups must also be addressed. However, the subordination of individuals to the benefit of the population (i.e. eugenics) must be resisted. Those engaged in genomics projects or diagnostics may need protection from hype and misuse of their personal data, There are different perspectives on the fair allocation of resources to healthcare and research for rare conditions. Health economics and health technology assessment can be practised equitably, so as to meet the challenges of rare disease clinical trials and address the needs of patients and communities.

## Background

This paper provides an overview of a workshop held in January 2025 on the theme of Justice in Rare Disease Genomics at the Fondation Brocher (Hermance, Geneva, Switzerland). Here we set out the context of the workshop, including its aims and scope, and present some of the broad issues discussed. Other papers in this collection look in more detail at specific topics.

## Introduction

Questions of justice arise in the implementation of genomics in clinical practice and research at many levels, especially for rare conditions. There are broad and general issues relating to justice that apply across the whole of healthcare, and there are other issues that apply more specifically in the context of genomics and of rare disease. The broader issues include questions of access to healthcare, participation in research, and the equitable allocation of resources; the narrower, more specific issues include, for example, the inference of personal or group identity from genetic data. The workshop aimed to consider the full spectrum of issues but with a focus on those more specific to rare diseases.

In the following brief sections, we outline some of the justice-related issues impacting medical genetics and genomics that have already received some attention. These points provide some background information.

### Population group diversity

One issue of concern regards diversity of population groups included in genomic data sets and research. The heavy concentration of genome-based research, and especially of population studies and biobanks, on people and populations in Europe and North America and/or of European descent has been acknowledged and first steps have been taken towards mitigating the problem. East Asia is now better represented in research, although that raises other issues, as international access to those data are often restricted. Efforts are being made to address the enormous genomic diversity in Africa, where much the greatest reservoir of human genome diversity is found (Ramsay et al. 2026). However, these efforts could be strengthened by further supporting local populations to become more involved in studying their genomes and applying the knowledge to the health of African peoples (Kamga et al. 2025).

Broadening the population base in genomic studies marks real progress but also presents a problem. It will be of value clinically in the interpretation of sequence variants of uncertain significance but the benefits of this will accrue primarily to those in privileged populations with good access to genomics in healthcare unless access to healthcare is transformed across under-served populations.

### Resource allocation for research into rare disorders and their treatments

Justice-related questions of resource allocation arise in an especially acute form in the context of rare conditions. While genomics has transformed our understanding of these conditions and enabled rapid growth in diagnostic precision, the question of fair allocation of resources to rare disease patients presents additional challenges not only to the patients and their families but also to physicians, researchers and pharmaceutical companies. If resources are to be allocated to a condition on the basis of the numbers of those affected, whether for research or for care, this might be tantamount to the abandonment of those with rare conditions. The justification for costly research into rare diseases must rely on additional considerations beyond simply the number of those affected. Indeed, the need for such research is arguably much greater than for many common conditions, whose disease mechanisms have already been at least partially elucidated, and for which some effective treatments have already been developed. However, there is a tension because the number of those likely to benefit from such investment is small. Furthermore, there are additional problems with clinical trials, when a possible rare disease treatment (‘orphan drug’) has been identified, because the numbers of patients available for trials of treatment are so few that the procedures to establish a trial and evaluate a treatment have to be somewhat relaxed.

### Healthcare challenges for rare conditions

There are many ways in which those affected by a rare disease may be at an added disadvantage in healthcare compared to others. Less will be known about their condition, even by one of the few experts in each particular condition, than with less unusual conditions. Furthermore, the rare disease can become a distraction from common ailments: a patient with a rare condition needs their physician to be aware of that condition and to look for possible links to any symptoms they have but it is unhelpful if all their health problems are immediately attributed to their (often poorly understood) rare condition. People with a rare condition develop common ailments too. The various challenges are summarised in ‘Equity for Rare’ (a report from Genetic Alliance UK, 2026) as ‘low priority, low clinical familiarity, low evidence’.

While it is inevitable that healthcare practitioners will often be ignorant about a patient’s rare condition, either because little is known or because they have not previously encountered it, there are additional factors that make it more difficult for these patients to access care. Those with chronic disabling conditions are often socially mobile ‘downwards’, finding it more difficult to succeed in education and obtain well-paid employment. If multiple family members are affected, the whole family may be impoverished (Burghel et al. 2020). In sum, these factors can impact their ability to afford basic self-care, to access disease-related information through the internet or to travel to regional hospitals and *a fortiori* to remote specialist clinics. These effects demonstrate the operation of Tudor Hart’s ‘Inverse Care Law’ (1971), that specifies that those in greatest need generally have the least access to health care (and the converse is also true). The level of understanding of their condition by a patient may also be limited if the condition impacts cognitive functioning.

### Diagnosis vs. Prediction

One major scientific challenge facing the application of genomics in medical practice, especially relating to rare conditions, is the asymmetrical relationship between knowledge of a patient’s condition (their phenotype) and knowledge of their genetic constitution (their genotype). From knowing that an apparently healthy fetus or child has a specific genetic variant – of genome sequence or of chromosomal structure – we may still be quite unable to predict what effect (if any) this variant is likely to have in their particular future. While patients do not have to wait so long to have a genome sequence analysis, so that those with a molecular diagnosis established have a much briefer ‘diagnostic Odyssey’ than would have been the case in the past, there is the different problem of the ‘phenotypic Odyssey’: the search for possible ill effects of a plausibly pathogenic gene variant in a (so far) healthy individual. The need for caution in the claims we make for genome-based predictions is clear. The need for modesty in the claims we make for gene-based treatments (gene therapy, base editing and the like) is stronger still.

Another aspect in the application of genomics is how this is described and presented to patients. Genomics is often thought of as ‘personalizing’ healthcare, in both diagnosis and treatment, and this might seem desirable for those with rare genetic diseases. However, it is possible for this to go too far. In the context of common conditions, there are collective, public health approaches to prevention and management – simple, cheap and effective - that are perhaps being sidelined in favour of the more expensive, less effective genomic approaches that lead to the individualization of responsibility for sickness. In the same way, we may need to consider how collective approaches to health can be used to the benefit of those with rare diseases: when are there collective approaches that can be of benefit to those with these conditions? More generally, when is genomics not the (sole) answer, and how do we promote genomics while, at the same time, retaining this broader perspective that recognizes the value of collective approaches?

### History

Another factor in the background to genomics, and that may have implications for how genomics could be implemented justly, is our history as a profession. As is well-known, this has been rather mixed. Problems have arisen when the wishes and well-being of individuals and families (as patients or participants in research, or both) have been subordinated to supposedly grander considerations, such as The Public Health, the People or Progress.

Consider eugenics, which in many countries (not only in Germany, 1933-45) subordinated individuals and families to some abstract notion of population health, riding roughshod over their interests, rights and even their lives. Is eugenics dead and buried? Well, there is no simple answer to that (Czech et al. 2023; Aylward et al. 2026). There are increasingly strong echoes of 20th century eugenics in some contemporary politics, such as in the white supremacy movement and Christian nationalism in USA and in some forms of populist politics more generally.

Having said all this, and acknowledging recent problems in human genetics research - such as the supposed ‘enhancement’ by gene editing of pre-implantation human embryos (Dimond et al. 2023) and the unauthorised release of participants’ data from research biobanks (Collins 2026) – there have been great efforts made by researchers in human genetics to define high standards of ethical conduct and to abide by them.

### Hype

Finally, there is the persistent problem of ‘hype’ in the announcements made at the launching of new genomics projects and the release of their findings that exaggerate the significance of any benefits, raising false hopes in patients and families, that then lead to disappointment among patients and their families. Such hype has different motivations in different contexts but there is often an element of seeking personal, institutional or corporate advantage in terms of money or status. There is a particular need for caution where genetics meets the new reproductive technologies. In such areas, inflated claims are rife and commercial applications may be unethical, being at best premature and quite possibly fraudulent, or at least delusional.

## Goals of the workshop

Given this background, we make no apology for considering how justice should be brought into discussions of rare disease genomics or assessing potential areas in which injustices may be occurring now or may be only too likely to arise in the future.

Developing genomics and integrating it into the clinic to benefit the patients and their families, requires awareness of these actual and potential problems. To warrant the enthusiasm for genomics, any abuse or exploitation of the disadvantaged must be identified and countered. Corrosive anti-science cynicism should be avoided while constructive criticism should be fostered.

While the issues of justice relating to rare disease genomics have begun to be addressed by those involved in genomics research, this process has been patchy and incomplete. A workshop to promote broader discussion and disseminate other perspectives seemed timely. We were fortunate in receiving support to hold a workshop on this topic at the Fondation Brocher, on which we will now report.

The first goal of the workshop was to develop **a consensus understanding of the problems faced by rare disease patients and families where justice is at stake**. Along the way we will need to consider the range of problems that the implementation of genomics could potentially exacerbate, if not handled well.

The second goal was to achieve **a shared perspective on support for rare disease patients**,** pooling our different disciplinary perspectives and lived experiences**. Elliott Mishler’s terms, the ‘Lifeworld’ and the ‘World of Medicine’, provide a useful frame to consider these (Mishler 1984). A contemporary perspective from the UK is that of the 2026 report, ‘Equity for Rare’, from the Genetic Alliance UK.

Our third goal was, through deeper engagement in the experience of both patients and practitioners, to consider **the implications for justice in several areas of rare disease genomics**, both in research and in its clinical implementation in healthcare. For example, we sought to consider the diagnosis of pre-disease states, the making of reproductive decisions about rare, inherited conditions, and the points of distinction between rare and common diseases.

Our final goal for the workshop was **to develop an informal network for future collaboration**. The two external groups who came together to initiate this project have a track record of contributing to the responsible progress of genomics and developing international networks. The Policy and Ethics Committee of the European Society of Human Genetics is expected to continue its functions indefinitely and the (UK-based) Medical Research Council’s Rare Disease Node on ethical and social aspects of rare diseases (led by Ramona Moldovan in Manchester) is being supported until summer 2028.

## Workshop contributions

### When a disease is rare: lived experience, multiple perspectives and the need for advocacy

The workshop started with a talk given by Sarah Wynn (rare disease advocacy, ‘Unique’, London, UK) on **Living with a rare chromosome or gene disorder.** She discussed the lived experiences of patients and families affected by rare chromosome and gene disorders, highlighting both the hopes and challenges. It has often taken many years for rare disease patients to be given a diagnosis but ending their diagnostic Odyssey is only the start of their journey with a specific diagnosis, for patients and families. ‘Unique’, a charity that provides support and information for families affected by rare chromosome and gene disorders, has surveyed families about their hopes for what genomics can bring and also attends to the challenges of daily life with or caring for someone with a chromosome or gene disorder. One key finding has been the need to manage the expectations of patients and families affected by rare conditions, to maintain trust and hope without building unrealistic expectations.

Sofia Douzgou Houge (clinical geneticist, Haukeland University Hospital, Bergen, Norway AND the Norwegian Centre for Rare Diseases, Oslo, Norway) gave her presentation, **Caring for rare disease: stories of walking through uncharted territory.** Sofia highlighted that rare conditions, with a point prevalence of < 5/10 000 (and < 1/50 000 for ultra rare conditions), together affect 3,5–5,9% of the global population, including 18–30 million persons in the EU, and most of these rare conditions have a genetic basis which is becoming more readily identifiable through genome-based diagnostic approaches.

Clinical care for those affected and their families is practiced at the rapidly changing intersection between these genome-based diagnostic technologies, web-accessible resources such as social media (including generative AI), and public sector participation in the financing of healthcare. Despite an appearance of systematic and coordinated approaches, the type or quality of care offered to each patient/family is very variable, often influenced by local access to expertise or to research and clinical trials and by individual factors (sociodemographic characteristics) rather than an approach based on evidence or on need. The key requirements for providing any sort of clinical care - i.e. listening to the patient’s questions and concerns, establishing a relationship with empathy, following up over time, offering regular communication and providing support for the affected person and the family - are often sidelined and not given their due weight. In fact, experience shows that the major, common diseases of developed countries are given priority in funding for both service developments and research. The flexibility that has permitted some small projects to support rare diseases in the past seems now to have been lost.

Sofia emphasised the importance of building an inclusive delineation of rare diseases, training healthcare professionals to care for those affected or at risk, raising social awareness of the ethical context of rare diseases, adopting public healthcare approaches that include the quantification of the value of care and not just its cost, and supporting research and services for rare diseases through public healthcare systems. This all needs to be worked for in association with patient groups to exert influence and leverage, to lobby governments and funding committees, and to attract private funds.

Nick Meade (Genetic Alliance UK) presented ‘**Looking at all rare conditions - ensuring equity between conditions’**. There are multiple dimensions of inequity between rare conditions. One is in the availability of a specific and effective treatment. Thus, only 132 of more than 7,000 rare conditions have a matching indication for a medicine authorised by the European Medicines Agency. This inequity in treatment availability, it was argued, arises largely because of inequity in research activity. A UK study (Rare Disease Research Landscape Steering Group and Bainbridge K. 2023) showed that less than 400 conditions had been the subject of a research award from a major funder in the UK between 2016 and 2021, while 23 of those had received 10 or more awards.

Genetic Alliance UK’s 2024 Rare Disease Day report, ‘Stats Behind the Stories’, examined the potential of studying a cohort of the most prevalent rare conditions as a lens to examine further dimensions of inequity. The report outlines multiple inconsistencies in the availability of services for this cohort, including access to commissioning pathways that should be reserved for conditions with much lower prevalences. Some of this inconsistency is due to intrinsic unfairness in decision-making pathways, which suffer (passively) from the scarcity of information about rare conditions. This passivity drives inequity by favouring conditions that have previously been selected for research or data collection or have been the lucky recipients of funding or a focus of research. In turn, this creates the necessity for complex advocacy from organisations who support people living with under-served rare conditions. Complex processes with multiple dimensions for evidence requirements, such as the Highly Specialised Services commissioning route and the UK National Screening Committee, further disadvantage these under-studied conditions with unrealistic expectations for quality of evidence.

Genetic Alliance UK argues that decision-making processes should be designed to be inclusive and to foster equity among diseases, not to allow rarity to be a barrier to services. System-wide approaches are needed for equity, as advocacy on an individual basis amplifies inequities. Some examples show equity and fairness to be feasible. Thus, the research pilot for genome-based newborn screening in England (the Generation Study) uses an active process for selecting conditions to be included that demonstrates how simpler criteria can be more accommodating in the face of gaps in the evidence base available for rare conditions. In addition, the UK Human Fertilisation and Embryology Authority (HFEA)’s process for deciding whether a condition should be licensed for preimplantation genetic diagnosis demonstrates how a threshold can be used to define eligibility without the necessity of more detailed quantification of complex metrics where evidence may be scarce.

Nadine Großmann (individual living with an ultra-rare condition, advocate and researcher, Freie Universität, Berlin, Germany) gave her presentation on **Rare Diseases: Lived Experience**,** Research**,** and Patient Advocacy**, in which she shared her personal journey with fibrodysplasia ossificans progressiva (FOP), an ultra-rare genetic condition that causes soft tissues to turn into bone. Her diagnostic journey was long and challenging, taking nearly 10 years to arrive at an accurate diagnosis. This delay not only impacted her health but also highlighted the struggles many individuals with rare diseases face in getting proper care and attention. Nadine reflected on the intersection between her lived experience and her advocacy work. As someone who lives with FOP, she is deeply committed to using her experiences to help others facing similar challenges, holding leadership roles within several patient organizations at the national and international level. These organizations are critical for raising awareness, providing resources, and creating the necessary connections between patients and caregivers.

Through patient advocacy, Nadine works not only to represent the voices of those living with rare diseases but also to advocate for better access to care, more funding for research, and necessary changes in policy. These roles have allowed her to help shape the global conversation about rare diseases, working alongside other patients, healthcare professionals, and researchers to create positive change. She emphasized the importance of ensuring that the lived experiences of people like her are central to the decisions that affect their lives. Alongside these advocacy efforts, she is also pursuing a Ph.D. researching FOP. Her personal experience with the disease informs her research, thereby contributing directly to the scientific understanding of FOP. Nadine hopes to uncover new insights that can lead to better treatments and, ultimately, a cure, but the research is not only about scientific discovery but also about improving the quality of life for those living with rare diseases like hers.

In conclusion, this talk aimed to show how the lived experience of rare disease patients is vital to both research and advocacy. The combination of personal experience, scientific inquiry, and patient advocacy is a powerful force for change, helping to create a future where patients are heard, understood, and supported.

Roseline Favresse (rare disease advocacy, EURORDIS) gave her presentation on **Building health and research literacy for rare disease patients and advocates.** She outlined how EURORDIS works systematically to ensure that the patient voice is meaningfully integrated into decisions affecting their health and care. This engagement spans multiple stages and levels of the healthcare and research ecosystem. EURORDIS promotes patient involvement from the early phases of research prioritisation and funding, through participation in Health Technology Assessment (HTA), to regulatory evaluation, approval, and ultimately access to rare disease treatments. These efforts align with broader European policy priorities and are actively supported by the European Union. However, despite this progress, significant disparities in access to treatments persist across Europe, exacerbating inequalities experienced by people living with rare diseases.

A cornerstone of EURORDIS’s capacity-building efforts is the EURORDIS Open Academy, which equips patient advocates with the knowledge and skills needed to engage effectively with healthcare professionals, regulators, policymakers, and industry stakeholders. The Academy offers both in-person and online training programmes, with materials available in multiple languages, thereby broadening accessibility and inclusivity. EURORDIS has also played a key role in embedding patient participation within the European Medicines Agency, supporting involvement across the full lifecycle of the evaluation and approval of medicines. In parallel, it strengthens patient engagement within the European Reference Networks by providing peer learning opportunities, practical tools, and ongoing support. Initiatives such as the Patient Partnership Hub further foster structured collaboration between patient communities and other stakeholders.

In the research domain, EURORDIS contributes to and supports patient involvement through initiatives such as the European Rare Diseases Research Alliance (ERDERA), reinforcing the role of patients as active partners in research design and implementation. More broadly, it advocates for the systematic inclusion of patient perspectives in decision-making on healthcare delivery and research. EURORDIS is also actively engaged in Screen4Care (S4C), an EU-funded initiative advancing newborn screening. This programme is working to define and expand the range of conditions included in screening programmes, with the aim of enabling earlier diagnosis and intervention for rare diseases.

### Approaches to justice

Angus Clarke (clinical geneticist, Cardiff University, Wales, UK) spoke on **The scope of social justice as it relates to genomics.** First, he mentioned two aspects of justice in relation to genomics that have already received some attention: access to healthcare, and the representation of different populations in research projects and population biobanks. He then outlined five additional areas that need to be considered in any assessment of justice within genomic healthcare:

- the use and possible misuse of genomic data,
- epigenetics, deprivation and the life-course (i.e. genetic factors in the developmental origins of health and disease across the life course),
- genetic testing for mental health and intelligence,
- the use of polygenic influences on disease to drive the individualization of responsibility for health, and.
- genetic screening and testing in reproduction.

Angus then considered the difficulties inherent in any unidimensional assessment of health outcomes, as required in strictly utilitarian approaches to the allocation of resources within healthcare. A much more promising account can be derived from John Rawls’s theoretical approach to justice as fairness, with his notion of the ‘original position’ of ignorance about one’s lot as the starting point for discussion. This is especially apt when considering the situation of those affected by rare diseases: how better to weigh their claims upon the community than by making decisions before we know who will be affected? The framework of Daniels and Sabin, which applies Rawlsian fairness to healthcare, allows rare diseases to be given their due weight in public discussion, while fully acknowledging the broader role of the social determinants of health (Daniels 2001).

Ruth Horn (bioethics, University of Augsburg, Germany) spoke on **The social**,** political and ethical context of rare disease genomics.** She pointed out that while much effort is being put into large-scale genomic sequencing programmes to detect and diagnose rare diseases and improve treatments, there are not only positive effects but also negative effects to which we ought to pay attention. It is important therefore to take a closer look at both the benefits and the possible disadvantages or harms resulting from genetic/genomic programmes at different levels: First, the broader societal implications of large genome programmes and their impact for public trust were discussed. Second, the meaning of fair and just allocation of public resources, based on considerations of the economic costs and benefits of genomic innovations, was examined. Third, the benefits of these innovations for stakeholders (clinicians, patients, and families) at the clinical level were investigated. Finally, the potential of genomics to improve health at the population level was explored. In conclusion, Ruth argued that for genomic programmes to be justified and gain public support and trust, the positive effects will have to outweigh the negative effects, and all patients must be able to perceive benefits.

To address possible negative effects, Ruth proposed a number of points that warrant particular attention: (1) (unrealistic) expectations of genomics that are not matched by its performance must be managed; (2) possible broader implications of testing, including the subsequent options that might emerge, must be communicated in good time, such as at the time of testing or when returning results; (3) costs and benefits, including individual and societal perspectives, must be assessed carefully; (4) social security safety nets must be put in place to avoid discrimination and stigmatisation; (5) equity and inclusion of underrepresented populations must be focused on; and finally, (6) political and policy choices must foster awareness of and address the negative effects of genomics (Horn et al. 2025).

Kelly Ormond (bioethics and genetic counselling, ETH Zürich, Switzerland) presented her draft paper on **Rare disease treatments and justice.** She started by arguing that the constructive uses made of genomics in society must be maximised while ensuring equity in how genomics and gene-based treatments are developed and applied. Challenges at present include the factors that lead a researcher to address one disease rather than another, such as the interest of a pharmaceutical company or the persuasion of the vocal, wealthy parent of an affected child. Access of patients to diagnostic technologies and to clinical trials is highly variable, often being complex and burdensome, including the requirement for long-term follow-up. Clinical trials are also potentially hazardous and geographical differences in access often reflect pre-existing and deep-rooted inequities. While a focus on the principles of distributive justice appears fair, Kelly highlighted the need to remember that the allocation of equal resources will often not lead to equity of outcomes.

Kelly and her team were in the process of conducting a scoping literature review, addressing the topic of health equity and justice around gene therapies and gene editing therapies; she reviewed the current status of the project. They had screened many articles and included 71 that served as the focus of the workshop presentation. They identified a series of potential themes: (1) The lack of operationalization about what equity and justice mean in the literature about gene therapies; (2) the large but generally unexamined topic of access to genomics, including its impact on diagnosis and hence on access to clinical trials, and also the impact of levels of knowledge (for the public, and also for referring doctors) on access. Ultimately, these effects all impact on access to therapies; (3) the recognition of cost as a core issue, and the one that is most written about, touching upon regulatory approvals and market approvals, Health Technology Assessment (HTA) frameworks, reimbursement and price transparency; (4) a smaller body of literature discusses the impact of translational access in LMICs, and globally in general; (5) other smaller topics include drug development processes, patient preferences and outcomes and racial disparities.

### Justice in health technology assessment and health economics

Elena Nicod (dolon.com, London) began this session with a presentation entitled “**Health economics of rare diseases”**, prepared by herself along with Mike Drummond. The central tenet in health economics is the consideration of opportunity costs, which examines the benefits of any treatments displaced, or not provided, when a new treatment is approved for funding. Cost-effectiveness analysis is the application of this concept and is frequently used by HTA bodies to inform resource allocation decisions by maximising overall health gained and minimising the opportunity costs (what is lost). Horizontal equity underpins this approach, where equal treatment is applied to all individuals regardless of the needs between individuals. The measures available to assess the effectiveness of rare disease treatments are patient-reported outcome measures, such as the EQ5D, which may not capture the full impacts of a disease or treatment, particularly in more severe and chronic conditions that are multi-morbid or heterogeneous. Given the small numbers of patients available for studies, it is difficult to generate evidence or to achieve economies of scale in research.

Applying this approach to rare diseases, many treatments would not be funded. Health economists and HTA bodies have acknowledged that there may be arguments for valuing some treatments more highly than others. Some of the arguments (e.g. severity of disease) apply to many treatments and do not alone justify the higher expenditure per patient for rare disease treatments. The argument for expenditure on rare disease treatments depends mainly on views about equity for individuals whose only effective treatment option is an expensive one, due to the rarity and innovative nature of the treatment.

The presentation of Victoria Charlton (King’s College, London), **Values underlying Health Technology Assessment**, focused on the values relevant to the assessment of technologies for rare diseases and the grounds on which the adoption of such technologies might be defended as morally justified. Taking it as a given that those responsible for conducting a HTA have good reason to want to show that their decisions are morally justified, Victoria suggested that an HTA body’s approach might be ethically evaluated in part based on its overall coherence; that is, based on the extent to which an HTA body’s values, principles, standards and case-based judgements are mutually supportive and warrant the judgements and recommendations that are made.

Using the UK’s National Institute for Health and Care Excellence (NICE) as an example, she demonstrated how technologies for rare diseases might pose a challenge to establishing such coherence, particularly when a decision-maker’s general approach relies on a utilitarian view of fairness and a commitment to a principle of cost-effectiveness. However, she proposed several alternative principles and values that might be successfully invoked to support the uptake of (often *cost-ineffective*) technologies for rare diseases. In particular, she highlighted that though rarity in itself may not be a morally salient consideration, those suffering from rare conditions often experience several significant and distinct sources of disadvantage which could justify the adoption of technologies that benefit these patients on prioritarian or sufficientarian grounds. Victoria emphasised that the strength of the moral argument for prioritising rare conditions will vary across cases and that those seeking to advocate for the adoption of rare disease technologies should be sensitive to the ways in which different cases are morally distinct, as well as to the ways in which they are similar. She concluded by arguing that, in all cases, given the very high prices charged for many of these technologies, claims for priority based on the value of ‘innovation’ should be subject to robust moral scrutiny and that close attention should be given to what can reasonably be accepted as a ‘fair price’.

The last presentation in this session was given by Hadewych Honné (PhD student in Science, Technology and Innovation Studies, University of Edinburgh and KU Leuven). In (**Re)defining ‘good’ care?: patient involvement in the valuation of an orphan medicine**, Hadewych presented HTA as a formulised or ‘formulated’ way of enacting solidarity by employing established pharmacoeconomic methods. In the case of extremely expensive (ultra) orphan medicines – which ‘stretch’ the principle of solidarity due to their high prices – the application of traditional pharmacoeconomic methods by HTA bodies often results in their non-reimbursement and thereby inaccessibility to patients. In recent years, however, HTA bodies have made attempts at altering pharmacoeconomic methods to account for the specificities of (ultra) orphan medicines, as well as relying on patient involvement to acquire greater understanding of the burden of rare diseases on patients and families, with the aim of enhancing the legitimacy and credibility of HTA.

Hadewych’s research interest is in how such patient involvement and input – often considered subjective or experiential and therefore considered as separate from the pharmacoeconomic assessment of medicines – actually interacts with formalised or formulated ‘registers of value’ (Heuts and Mol 2013) in HTA. She presented a case study from her PhD research, focusing on the evaluation of Soliris for reimbursement in atypical Haemolytic Uraemic Syndrome (aHUS) by the national HTA body of the Netherlands. She showed how the Dutch national aHUS patient organisation collaborated with clinicians to show that Soliris – which had been marketed as a lifelong biweekly treatment to inhibit uncontrolled complement system activation in aHUS patients – could be used in a tailored way in individual patients. Many patients, particularly those with a specific gene mutation, could cease treatment altogether, thereby drastically reducing the volume of Soliris needed to effectively treat patients.

The patient organisation’s input into the assessment procedure by the Dutch HTA body emphasised the importance of increased patient comfort (by virtue of a much less intense treatment approach) and safety (by preventing risks of meningococcal infections) resulting from treating aHUS patients in line with the ‘Dutch treatment guideline.’ Moreover, the organisation emphasised the solidarity shown by aHUS patients in voluntarily taking part in research into the tailored use of Soliris in aHUS, thereby taking the risk of their aHUS relapsing. In doing so, the patient organisation raised uncertainty around the ‘real world’ effectiveness and cost-effectiveness of Soliris as compared to the manufacturer’s clinical trials. Hadewych argued that the patient organisation’s submission thereby engaged with the values of necessity, effectiveness and cost-effectiveness as formulated by the Dutch HTA body. She argued that ‘subjective’ and ‘experiential’ input from patients is intricately entangled with formalised and formulated pharmacoeconomic assessments in HTA, and that the two should be considered in conjunction, rather than being separate components of the HTA process.

### Representation of population groups and other communities in genomics research and databases

Ambroise Wonkam (Genetic Medicine, Johns Hopkins University, Baltimore, USA) presented on **Harnessing our Common African Genomes to Improve Health Globally.** He made the case for studying African genomic variation in terms of ancestry, ecology, and equity. While Africa is the birthplace of modern humans, and hosts the bulk of human genetic diversity, fewer than 2% of human genomes analysed to date have been from individuals of African ancestry. As Africa is home to millions of unique genetic variants and, on average, genomes of African ancestry contain three to five times more variants than those from other populations, studying African genomic variation is now clearly a scientific imperative.

Ambroise emphasized the importance of African ancestry in genomics. All modern humans share a common ancestry, with approximately 5%–15% of our genetic heritage tracing back to lineages that diverged as far back as 1–3 million years ago, before the original migration out-of-Africa, but with multiple instances of genetic exchange between populations in both directions before this and since. Furthermore, African ecology needs to be taken into account. The wide variety of climates, cultures, languages, and ecosystems across Africa has led to ecological pressures shaping genetic variation in different ways across different African population groups. Many novel variants have arisen which are likely to be functionally important and could therefore have implications for genetic medicine. Hence, to foster equity in genomics, sequencing millions of African genomes is essential to addressing health disparities, particularly in relation to genetic diseases that disproportionately affect African populations, such as several haemoglobinopathies, and rare and undiagnosed diseases in general. For example, identifying previously unknown genetic risk factors for the complications of sickle cell disease (SCD) could prove most helpful. Expanding genomic research in African populations will not only improve diagnoses in Africa but will also provide broader insights into the underlying causes of rare and complex diseases.

In conclusion Ambroise argued that increasing the availability of African genomes will enhance our understanding of human evolutionary history, improve studies on genomic variation linked to complex traits, and help refine genetic medicine for Africans and non-Africans, by improving the interpretation of genetic variants. By investing in research on African genomes, not only knowledge of human genetics will be advanced but also work toward more equitable healthcare solutions, benefiting African populations and the world at large.

Angeline Ferdinand (Centre for Health Policy, University of Melbourne, Australia) spoke on **Bringing Justice for Marginalised Groups into Clinical Care for Rare Diseases—An Australian Perspective.** Advances in genomics offer opportunities to improve clinical care, yet equitable access to genetic services remains a challenge for marginalised populations. In Australia, Aboriginal and Torres Strait Islander peoples (3–4% of the population) have high fertility rates and experience significant unmet needs in healthcare generally, such as late diagnoses and poor outcomes for cancer. There are also unmet needs specifically in clinical genetics and genetic counselling, with substantial gaps in service availability, affecting both the comprehensiveness and the continuity of care. Addressing these disparities is critical to ensuring fair and effective health care access.

The 2020 Australian Rare Disease Action Plan proposes a person-centred, action-oriented and sustainable approach encompassing health care and education. However, while equity is recognized as a strategic priority in Australia, systemic barriers persist, including poor data quality (especially for minority population groups), inadequate clinic space for family groups to be seen, and poor provision of follow-up care. High-quality, disaggregated data are essential for investigating health disparities and guiding targeted interventions. However, clinical genetic data infrastructure exemplifies embedded issues: Indigenous status is missing for ~ 35% of patients and inconsistencies in clinic identifiers obscure patient pathways. These limitations hinder the ability to assess and address gaps in service provision effectively. Practical, physical constraints on service facilities render them less appropriate for those from the more vulnerable groups.

Given the well-documented disparities in health care access for Aboriginal and Torres Strait Islander peoples and culturally and linguistically diverse communities, improving genetic service delivery will require better data governance, its purposive alignment with these policies, and meaningful engagement with these populations.

Salman Kirmani (medical genetics and paediatric endocrinology, Aga Khan University, Karachi, Pakistan) presented his paper on **“Current Status and Future Needs of Services for Rare Diseases in Pakistan”.** Low-Middle Income Countries (LMICs) like Pakistan are burdened with large populations and poor public health infrastructure. Given the high rates of consanguineous unions in these countries, the number of patients with rare diseases is far greater than in better-resourced nations, creating huge unmet needs for families navigating an already over-burdened health system. There are few trained healthcare providers and few diagnostic services for such families, and the exorbitant cost of precision therapies has created a huge access gap in the global south. The low level of education and awareness also magnifies problems such as fatalism about the course of disease, the common practice of mothers being blamed for genetic conditions affecting their children, the stigmatization of family members when a genetic or inherited condition is suspected, and the high frequency of consanguineous marriages (more than half of marriages being between cousins).

Thus the issues of justice are magnified in low-resource countries, and the genomics community must learn to advocate for global equity in rare diseases, so that novel breakthroughs in precision medicine can also benefit those patients who are in the greatest - the most dire - need for such services.

### Protecting patients in rare disease research

David Freis (History of medicine, University of Augsburg, Germany) drew attention to the past – specifically to the abuses of medicine and biomedical research in Nazi Germany – in his presentation, **Historical perspective on the medical abuse of patients.**

In present-day debates about ethics in medicine, historical examples and comparisons play a major role. Past abuses of patients are used to justify ethical codes and regulations for future research. However, this is often done in a way that reduces complex historical events to catchphrases obscuring the specific ethical implications of concrete historical events. This is most obvious in simplistic claims that “the Holocaust” somehow led directly to modern bioethics, conflating a broad range of Nazi crimes into a single concept, despite many of these crimes having little to do with either medicine or medical ethics. In this talk, the current state of research into the history of Nazi medical crimes was presented with a focus on four major topics: the expulsion of physicians for ‘racial‘ and political reasons after 1933, forced sterilisations on eugenic grounds, the mass murder of sick and disabled people in various ‘euthanasia‘ programs, and the inhumane experiments conducted on inmates of concentration and extermination camps. The use of historical arguments in ethical discourse should be based on a nuanced understanding of detailed, carefully differentiated, historical research.

A very different risk taken by participants in current genomic research is that of the inappropriate disclosure of their medical and/or genomic information held by the research team, perhaps to insurance companies or other agencies. Accordingly, Teodora Lalova-Spinks (KU Leuven and Ghent University) presented her paper on **A legal perspective on biomedical data sharing in Europe**.

Health research relies on the use and reuse of special categories of personal data, including data about health and genetics. Several types of research, particularly rare disease research, are heavily dependent on the exchange of data between countries. However, the current legal framework for the conduct of (cross-border) health research in the European Union (EU) is complex and diverse, being specific to each country’s legal system. The talk aimed to introduce the audience to the main applicable current and upcoming laws in the field, and to highlight examples of challenges related to the laws.

The legal framework was first described from a birds-eye view: namely, the complex interplay of several laws in the EU, such as the EU General Data Protection Regulation (GDPR), the Clinical Trials Regulation 536/2014, the Medical Devices Regulation 2017/745 and the In vitro diagnostic medical devices Regulation 2017/746. In addition, some national legislation has not yet been harmonized across the 27 EU Member States, specifically in the fields of research biobanking and genetic testing. Moreover, the EU has been active in introducing new legal and policy initiatives that will have an impact on research, such as the Data Governance Act, the Data Act, the AI act, and the European Health Data Space regulation (EHDSR).

Several challenges related to the GDPR were discussed, for example the rules pertaining to international transfers of (health) data (Lalova-Spinks et al. 2024). It was shown that the biggest hurdle remains the lack of harmonization in the case of cross-border research, which is linked to three main reasons: (1) the divergent national implementation of the GDPR, (2) the unharmonized interpretation of the GDPR, and (3) the contradictory advice issued by ethics committees on such matters in certain specific cases (Lalova-Spinks et al. 2022).

Difficulties in the cross-border sharing of data for research may have been exacerbated by the GDPR but Teodora moved to discuss how it is hoped that the EHDSR may ease these difficulties. Finally, Teodora shared information about a then-ongoing empirical study conducted in the scope of the Belgian ‘Symphony of Us’ project.^1^ This study gathered insights about the knowledge and critical reflections of key oncology research stakeholders (such as legal experts working with pharmaceutical companies, hospitals, research institutes; healthcare professionals; policymakers; ethics committees; patients and patient representatives) on the (re)use and governance of health data for research, and their understanding of the notion “patient value”. The study’s findings underscored that the legitimacy of the EHDS will depend on embedding patient values into its implementation through transparency, participatory data governance, and legal and technical safeguards (Lalova-Spinks et al. 2026).

The talk led into a discussion on the workshop participants’ experiences with legal and ethical challenges around data sharing and research collaborations; experience with the ways to navigate such challenges; and views on the role of stakeholder representatives (such as patients) in data governance.

Meghan Halley (bioethics and medical anthropology, Stanford Center for Biomedical Ethics) gave her presentation, **When Equity Is Rare: Advocacy**,** Equity and Rare Disease in the United States.** The scientific strides made in rare disease therapeutics over the last decade have generated justifiable excitement in the patient community, and the flurry of both public and private investment in rare disease therapies has been driven in large part by patient advocacy groups. The lack of market incentives for rare disease drug development in the United States means that support group advocacy is very important. However, these developments also have raised unique challenges arising at the intersection of equity and advocacy.

A primary focus among rare disease advocates, researchers, and drug developers has been on biases in research funding allocation, drug development, regulatory policy, and drug reimbursement practices that fail to account for the unique challenges of rare diseases. Advocacy by patients, their families, and their partners in academia, industry and government has been a primary lever applied to mitigate these biases and has resulted in a range of policy initiatives that create incentives for developing rare disease therapies. However, while advocacy can promote drug development for rare diseases, it also has the potential to drive disparities in terms of the distribution of scarce research resources across rare disease patient communities. Further, the high prices of these therapies also risk disparities in access to the benefits of innovations in therapies for a given rare disease, due to socioeconomic status as well as biological factors and genetic ancestry. While advancing therapies for paediatric diseases with high morbidity and mortality is arguably itself an ethical imperative, it is important to look more broadly. Without attention paid to the multiple intersecting dimensions of equity in rare diseases, the incredible advances underway risk widening the gap between those who can benefit from these innovations and those who cannot.

### Experience with rare diseases in Europe: care, research and advocacy

Elena Avram (Ophthalmogenetics, Bucharest, Romania) presented her **Review of rare disease national programmes in Europe.** As previously noted, rare or orphan diseases are not collectively rare: they are collectively common and represent a challenge for the public health system. Diagnosis and treatment are complex and require involvement of multiple healthcare providers. Many European countries have developed national strategies, programmes or plans to improve the care, diagnosis and treatment of rare diseases. The objective of this presentation was to review the principal national structured frameworks for rare diseases in Europe from 2005 to 2024. Multiple national strategies, programs and plans were identified: EU countries have a better outcome compared with many non-EU countries. However, many of the programmes identified have been time-limited and there are substantial differences in their continued support across Europe. Addressing rare diseases through comprehensive national plans will be crucial to provide equitable care across Europe.

Siddharth Banka (medical genetics, University of Manchester, UK) presented on **The Manchester Rare Disease Centre Research**,** Clinical Trials and Services** (https://www.mrcc.org.uk/*).* This presentation highlighted the establishment of a dedicated Centre for rare diseases. It began by outlining the recent history and policy context that shaped its development, and then detailed the Centre’s strategic framework, structure, and intended impact. Its four core themes are (i) to find a diagnosis for rare disease patients, (ii) to raise awareness of rare diseases among health care practitioners, (iii) to provide care coordination for rare disease patients, and (iv) to provide access to specialist care and treatment. The key aims and objectives of these four core themes, along with the contributions of the patient and public involvement group, were summarized. Finally, example projects showcased the Centre’s efforts in advancing research, clinical trials, and patient-focused services in the rare disease domain.

Charlotta Ingvoldstad-Malmgren (genetic counselling, Center for Rare diseases, Karolinska University hospital, Stockholm, Sweden) presented on **Rare disease experience in Sweden.** She discussed how research collaborations worked together with the patient representatives for rare diseases in Sweden. Researchers work with patients to develop questionnaires that explore and report the patient perspective. They also collaborate on how researchers and clinicians can use the patient surveys from “Rare Diseases Sweden” (from the past and into the future) to assess patient responses as a register of research data. Charlotta also showed how researchers collaborate with patient organisations to invite participants to take part in research surveys.

### Genomic testing, reproduction and identity

Felicity Boardman (social sciences, Warwick University, UK), gave her presentation remotely, on **Prenatal and neonatal genomic screening: issues of identity and uncertainty.** As genomic technologies move from clinical care into the domain of public health (e.g. the Generation Study, UK), ambiguous or uncertain results following participation in screening programmes are about to become more common. This means that increasing numbers of people will be living with chronic health risk and uncertainty as ‘patients-in-waiting’.

Research with families whose child was designated CFSPID (cystic fibrosis screen positive inconclusive diagnosis) through newborn screening demonstrates the various ways that these types of results can challenge the boundaries of health and illness, with impact on the sense of self, on the sense of belonging and on relationships (Boardman & Clark, 2022 – which formed the basis of this presentation). The concept of ‘genetic nomadism’ captures the oscillations experienced by these parents of children with CFSPID between the two ‘worlds’ of health and illness, whilst largely feeling displaced from both. Further support of families living with uncertainty and risk is now particularly important given that the unique challenges they face in terms of disclosure of information to the maturing child, surveillance and managing seemingly contradictory health messages are likely to become so much more common.

Hilde Van Esch (clinical genetics, KU Leuven, Belgium) presented her paper, **Perspectives on the newer reproductive genetic technologies: population carrier screening**,** pre-implantation genetic diagnosis – PGD - and non-invasive prenatal genetic testing – NIPT.** The reproductive field is rapidly expanding due to advances in genome-based technologies, and this talk addressed reproductive genetic carrier screening, preimplantation genetic testing and non-invasive prenatal testing. Current practice in Belgium was used as an example.

Through carrier screening, couples at-risk of conceiving a child with an autosomal recessive or X-linked condition can be identified prior to conception. In Belgium, a large exome-based gene panel has been offered since 2019 consisting of ~ 1600 recessive and ~ 100 X-linked disorders. Analysis for the FMR1 expansion associated with fragile X syndrome, and for spinal muscular atrophy (SMA) and for Duchenne muscular dystrophy (by MLPA) are added to the panel test. This test is made available for those able to pay out-of-pocket. Those at higher risk, such as consanguineous couples, more often do not take the test because of financial and/or cultural reasons. Another difficult point is determining the criteria for including a gene in the list. A national working group aims to monitor these criteria and the list of genes and of specific pathogenic variants (those to be reported) is frequently updated.

Preimplantation genetic testing is an established technology that is used to test embryos for monogenic/single gene disorders (PGT-M) or for structural chromosomal rearrangements (PGT-SR) present in one of the future parents. More recently also PGT-A, that screens for aneuploidies, has been implemented. In Belgium, evaluation of the PGT requests generally happens at the level of each PGT centre, embedded within a university hospital, and often in consultation with the local clinical ethics committee. In order to illustrate the ethical challenges in PGT, several clinical examples were presented. Questions arise about which conditions are severe enough for PGT to be allowed, or warranted, and how to make decisions about conditions with variable penetrance. On the other hand, can a couple with a high risk of passing on a Mendelian disorder to a child be obliged to include PGT if they need to conceive via assisted reproductive technologies (ART)?

Finally, NIPT was addressed. Technological developments have led to the increasing number of conditions that can be detected via NIPT (e.g. microdeletions, rare autosomal trisomies, rare CNVs etc.) in the fetus and/or mother. This presents some challenges, necessitating additional resources for pre- and post-test counselling and a more uniform approach to NIPT testing.

Tamar Nov-Klaiman (genetic counselling, bioethics, University of Augsburg) gave her presentation on **More of the same? Israel’s expanded carrier screening for cystic fibrosis.**

Most countries where cystic fibrosis (CF) is highly prevalent offer CF testing as part of newborn screening (NBS) programs to enable early diagnosis, timely treatment, and improved health outcomes. Israel, however, has adopted a distinct approach. Rather than offering CF testing via NBS, it has, since 2008, implemented publicly funded population-based carrier screening. Couples identified as carriers and therefore at increased risk of having an affected child are eligible for funded prenatal testing or in vitro fertilization with preimplantation genetic testing. The Israeli molecular panel for CF has expanded substantially from 18 to ~ 70 pathogenic variants. Tamar presented a project she is involved in, led by Ruth Horn (Augsburg) and Aviad Raz (Ben-Gurion University of the Negev). The project examines the ethical, social, and policy implications of this expansion, particularly in relation to NBS and in the context of emerging, effective but costly therapies. These treatments introduce a potential policy tension: on the one hand, their clinical benefits arguably incentivize a policy of treatment rather than the prevention of cases through selective reproduction; on the other, in countries with universal healthcare that focus on rationing resources, they might incentivize policies that facilitate prevention of cases through carrier screening, to decrease the number of individuals with CF that would require lifelong treatment and burden the healthcare system.

The Israeli policy broadens women’s reproductive options. However, the expected lower CF birth rate following the implementation of the expanded carrier screening panel weakens the drive to implement NBS. This raises ethical concerns, as individuals born with CF may be less likely to benefit from early diagnosis. Given that the expanded panel does not capture all pathogenic variants and that population uptake remains incomplete, carrier screening cannot identify all cases. In this sense, NBS and carrier screening function in a complementary way: together, they support both an expanded range of reproductive options and improved clinical outcomes for those born with the condition (Nov-Klaiman et al. 2025).

### Tackling injustice

The presentation of Sasha Henriques (Genomics England, London, UK) on **‘Returning to (the theme of) Justice’** explored approaches to addressing the persistent underrepresentation of some of the world’s most genetically diverse populations in genomic research. First, it was argued that the widening gap in data between European and non-European groups highlights inconsistencies in population categorisation and dataset construction, raising questions about how these practices contribute to inequities. Early findings from an ongoing ethnographic study at the Wellcome Sanger Institute have addressed how efforts to improve diversity in genomic datasets often overlook the structural and epistemological challenges embedded in race, ethnicity, and genetic ancestry classifications.

Grounded in Critical Race Theory, this study first examined the ethical dimensions of genomic categorisation, drawing on the report ‘Using Population Descriptors in Genetics and Genomics Research’ (National Academies of Sciences, Engineering, and Medicine, 2023) to highlight the tension between scientific objectivity and social justice. It reinforced how race and ethnicity are inconsistently applied in genomic research, challenging the assumption that avoiding racial categorisation eliminates bias. Instead, it proposed that addressing these disparities requires a critical engagement with how populations are defined, analysed, and represented.

Preliminary observations from the ethnographic research highlighted the fluidity of labels used to describe dimensions of genetic similarity, ancestry, race, and ethnicity across multiple data sources and scientific domains. This was observed despite strong support for genetic ancestry as a robust concept for categorising the human population. The presentation also discussed the limitations of diversity initiatives focused solely on sample recruitment and examined how power dynamics in genomic research shape the priorities that enable novel approaches to use more equitable methodologies. Funding constraints and institutional hierarchies, unsurprisingly, influence researchers’ perceived ability to challenge existing practices, reinforcing systemic inequities.

The presentation concluded with reflections on embedding social justice within genomic research, advocating a shift from treating diversity as a problem to integrating equity as a core principle. Researchers should be supported in developing socially just methodologies, suggesting that the convergence of interests could be explored as a viable approach to advancing racial equity while maintaining the importance of institutional accountability and commitment to structural change. Sasha emphasised that achieving equity in genomics requires broader representation and a critical re-examination of the frameworks underpinning data collection and analysis. Furthermore, the presentation highlighted the necessity of a more detailed discussion on the limitations of existing data usage. Finally, it underscored the opportunity for genomic research to lead the way in rethinking how populations are categorised and represented, ultimately driving more equitable data collection and use.

Ilaria Galasso presented her paper – the second one which had to be given remotely – on **Genomics and global justice: which benefits for socioeconomically deprived populations?** She questioned the benefit of genomic medicine for socio-economically disadvantaged, marginalized or historically exploited populations. In this regard, building on the documentary analysis and interview studies she had conducted for her doctoral and postdoctoral research, she examined three issues:

1. Can disadvantaged people *access* the benefits of genomic medicine?
2. Does genomic medicine *respect* disadvantaged people´s sovereignty?
3. Is genomic medicine what disadvantaged people *need*?

Regarding the question of *access*, Ilaria distinguished between access to participation in genomic research studies and access to genomic healthcare and argued that efforts for including disadvantaged people in the research do not contribute to health equity if the resulting therapies remain inaccessible to them (Galasso 2024). While the inclusion of disadvantaged populations in genomic research is essential for scientific progress and for making genetic health care at least in principle equitably applicable, it only exacerbates health inequalities if the issue of access to health care for these populations is not adequately addressed.

Regarding the *respect for people’s sovereignty*, minorities and vulnerable, marginalized or historically exploited populations have often been concerned with historical and contemporary controversies about genetic data (mis)use. In contexts of vulnerability, informed consent is essential but not sufficient: participant consultation and engagement, and transparency about the expected benefits are also required. A further step to minimize the risk for exploitation and the violation of sovereignty is the inclusion of community representatives in the research leadership, and – crucially - this requires active investment in capacity building (Galasso and Geiger 2025).

Regarding the issue of *need*, investments in genomic medicine will often contrast and even compete with the (unaddressed) lack of basic healthcare services for vulnerable groups, and with poverty-related diseases requiring simple public health and social interventions that are already well-recognized. Importantly, however, genomic medicine and public health are interrelated and, provided that genomic investments do not come at the expense of public health investments, genomics may provide a route to a better understanding of public health and poverty-related diseases and, ultimately, also to societal interventions.

Barbara Prainsack presented her paper on **Solidarity and Justice: What is the difference**,** and how do they matter for personalised medicine?** Her talk focused on different conceptualisations of solidarity, and the difference between solidarity and justice. In the second part of the talk, by introducing the notion of data solidarity, discussing how solidarity can give guidance for data governance (Prainsack et al. 2022; Prainsack and El-Saayed 2023). Further information about *data solidarity* from the Data Transformations for Health Lab can also be accessed via the Data Transformations for Health Lab website: https://dthlab.org/data-solidarity-glossary/.

Specifically, Barbara suggested that solidarity is best defined as a practice by which people support others to whom they consider themselves as connected in a relevant manner – such as a shared goal or a shared experience of injustice. Solidarity can take place at three levels: between two individuals (tier 1), within groups (tier 2), or at the level of formal institutions (tier 3). Barbara argued against a view that considers solidarity as merely ancillary or subservient to justice. Solidarity and justice are complementary, each capturing aspects and elements that the other one does not. For example, solidarity can help as a means to identify and address injustices, and justice provides normative guidance to solidarity (Prainsack 2026).

The workshop’s final session then addressed, “What Next?” in a general discussion on **Moving forwards … planning outputs from the workshop.** In this session, there was a general discussion about (i) the messages we would take away from the workshop, (ii) how to work on the outputs from the workshop and (iii) what future activities we could plan in support of justice in the implementation of rare disease genomics. Here, we focus on some of the take-away messages.

In relation to the workshop’s first goal, to *develop a consensus understanding of the problems faced by rare disease patients and families where justice is at stake*, we need to consider the range of problems that the implementation of genomics could potentially exacerbate, if not handled well:

- It is important to develop an awareness on the many and varied ways that injustices can arise in the context of genomics. Training to orient our thinking to identify now injustices that may not otherwise be recognized for some decades will be required. Identifying today’s parallels with the abuse of patients and minorities in the past would be a first step.
- Active inclusion of underserved groups in research and delivery of services should be prioritised.
- Africa is at the heart of human genetics and increased sequencing of African populations is essential to addressing health disparities and to improving health globally.
- Vast global challenges still exist from the legacy of colonial exploitation (in Australia, Africa, South Asia and the Americas).
- The research and clinical agendas often do not take (in)equity sufficiently into account.
- Data sharing is essential for research, but it can cause harm to patients, beyond simple identification. Risks should be carefully considered and addressed, particularly if the rules around data sharing are relaxed.
- Engagement in clinical trials is burdensome and can be hazardous. It will be important to avoid assumptions of benefit - the therapeutic misconception (Woods et al. 2014), and to offer recruitment in a balanced way.
- We need to manage expectations carefully, not to exploit people’s hopes, and thereby to maintain trust.
- Over-medicalisation leads to excessive interventions and therefore excessive risks.
- Awareness of the multiple dimensions of equity – the intersectionality - is often lacking. There is potential for inequities within diseases, between diseases, and between population groups as well as the more widely recognized inequities relating to sex, age, wealth and social class.
- The classification of people with rare conditions needs to be inclusive, so that no-one is left out.

Our second goal, *to achieve a* ***shared perspective on support*** *for rare disease patients by pooling our different disciplinary perspectives and lived experiences*, led to these messages:

- Rare disease patients need professionals to deliver care with a sensitive intelligence, with technical competence and with passionate advocacy (it matters how we talk about people and to people). These professionals need training if they are to nurture a ‘rare disease mindset’.
- Patient groups should be offered training, to support their representatives and enable them to make their full contribution.
- Consideration must be given to the fact that patient groups are fragmented and sometimes might be in conflict.

Our third goal was, *through deeper engagement in the experience of both patients and practitioners*,* to* ***consider the implications for justice*** *in several areas of rare disease genomics*, ***both in research and in its clinical implementation in healthcare***, leading to these messages:

- The diagnosis of pre-disease states:
  - Diagnoses can come late (be ‘delayed’) but they can also come too soon, especially if prognostication introduces great uncertainty.
  - Uncosted outcomes of genomic testing include the search for manifestations of a plausibly pathogenic gene variant, perhaps with much medicalisation and numerous investigations over many years – the phenotypic Odyssey.
- Making reproductive decisions about rare, inherited conditions.
  - The goals for reproductive genetic screening programmes are difficult to define: neither prevention nor autonomy is convincing as a primary goal to drive a programme and against which it can be audited.
- Re-considering the points of distinction between rare and common diseases:
  - In assessing outcomes, one needs to recognise context, including disease frequency, severity, hope, unmet needs, stigma, the range of management options, geography, implications for family members and reproduction.
- System-wide approaches are needed.
- National rare disease plans can be key tools in promoting just healthcare delivery and should be regularly reviewed to ensure they are comprehensive and remain appropriate.
- Expect coherence across a priority-setter’s judgements (e.g. sufficiency vs. priorities).
- It is essential to reach a fair price for drugs and treatments for rare diseases.

In addition to a report to the Fondation Brocher, the outputs from the workshop consist principally of this collection of related articles that include material delivered at the workshop but often developed subsequently. We hope these articles will stimulate not only further thought but also constructive action that will influence the phenomena they describe.

Finally, the fourth goal of developing an informal network for future collaboration. It is too soon to know if this will be effective but the publication of the full set of papers being developed by contributors to the workshop, over the next 6–12 months, will give an opportunity to assess this in early 2027.

## Data Availability

No datasets were generated or analysed during the current study.

## Footnotes

1 For more information: https://www.symphonyofus.be/en/?lang=en.
